# Retrospective Analysis of Human Papillomavirus Genotyping and Cytology (Pap Smears) in Cervical Cancer Screening: An Institutional Experience in the State of Oregon, USA

**DOI:** 10.3390/diagnostics15040419

**Published:** 2025-02-09

**Authors:** Zhengchun Lu, Maxwell Knapp, Siouxzanna Downs, Rabeka A. Ali, Terry K. Morgan, Heather M. Ruff, Xuan Qin, Guang Fan

**Affiliations:** 1 Department of Pathology & Laboratory Medicine, Oregon Health & Science University, Portland, OR 97239, USA; luzh@ohsu.edu (Z.L.); knappma@ohsu.edu (M.K.); alir@ohsu.edu (R.A.A.); morgante@ohsu.edu (T.K.M.); ruffh@ohsu.edu (H.M.R.); qinxu@ohsu.edu (X.Q.); 2 Office of Advanced Analytics, Oregon Health & Science University, Portland, OR 97239, USA; downssi@ohsu.edu

**Keywords:** HPV genotyping, HPV 16/18, non-HPV 16/18, high-risk HPV, pap smear, cervical cancer screening

## Abstract

**Background/Objectives:** The 2020 American Cancer Society guidelines endorse human papillomavirus (HPV) testing as the preferred method for cervical cancer screening. This study aims to evaluate the concordance of HPV and cytology findings for cervical intraepithelial neoplasia (CIN) at a population level. **Methods:** A retrospective cohort review of cervical cytology, HPV testing, and biopsies for all patients at a single Oregon-based medical center was performed over 21 months. The performance of HPV and cytology in detecting high-grade CIN lesions was compared. **Results:** A total of 22,488 tests were evaluated, showing 7.5% abnormal cytology and 7.4% positive HPV. Among 574 patients who underwent co-testing and a subsequent biopsy, 345 had abnormal cytology, with 212 having abnormal biopsy results. HPV was positive in 455 cases, with 266 having abnormal biopsy results. Among 455 HPV-positive cases, there were 283, 104 and 33 cases of non-16/18, 16, and 18 types, respectively. Additionally, 35 cases had co-infection with multiple HPV types. Among the cases diagnosed as CIN3 on biopsy, 90.6% had positive HPV testing (*N* = 96), and 82.9% had abnormal cytology (*N* = 94). HPV testing demonstrated a slightly higher sensitivity (88.8% vs. 78.3%, *p* = 0.128) and lower specificity (27.1% vs. 48.9%, *p* < 0.01) compared to cytology for CIN2 and CIN3 diagnosis. **Conclusions:** HPV testing showed a similar sensitivity but lower specificity compared to cytology for detecting high-grade lesions.

## 1. Introduction

In the United States in 2020, approximately 13,800 cases of invasive cervical cancer were diagnosed, resulting in an estimated 4290 deaths [1]. Widespread screening practices initiated in the 1950s significantly decreased the incidence and mortality rates of cervical cancer. The current 2020 guidelines for cervical cancer screening by the American Cancer Society (ACS) recommend a human papillomavirus (HPV) test alone every 5 years for everyone with a cervix from age 25 until age 65 [2]. While the primary HPV testing is the preferred method, co-testing and cytology (Pap test) alone is acceptable and people can get screened with an HPV/Pap co-test every 5 years or a Pap test every 3 years. Similarly, the 2019 American Society for Colposcopy and Cervical Pathology (ASCCP) guidelines describe best practices for evaluating cervical cancer risk. The results of HPV testing and cytology studies are integrated with patient history to determine a calculated risk of CIN3+. Using this risk, clinicians can determine an appropriate screening interval and evaluate if colposcopy is indicated [3]. The current ASCCP guidelines recommend one of six clinical actions (treatment, optional treatment or colposcopy/biopsy, colposcopy/biopsy, 1-year surveillance, 3-year surveillance, and 5-year return to regular screening) for the management of cervical cancer screening. This recommendation is based on a patient’s risk of CIN3+, considering both current results and past history, including unknown history [3]. These advancements adapt prior screening strategies to leverage the higher sensitivity of HPV testing in cervical cancer prevention [4,5].

The association between HPV infection and cervical cancer development is a dynamic process. HPV infection is common in healthy individuals, with most infections being asymptomatic. Only a small percentage of infections persist and progress to a precancerous state, including cervical intraepithelial neoplasia (CIN) and adenocarcinoma in situ (AIS) [6,7,8]. The progression to a precancerous lesion takes many years. Importantly, patients frequently clear HPV infection without disease-modifying intervention. In individuals under 25, as many as 44% of patients may spontaneously regress [9]. Unfortunately, many patients do not spontaneously clear infection, which increases the risk of developing carcinoma. A persistent infection with high-risk HPV (hrHPV), including 16, 18, 31, 33, 35, 52, and 58, is the cause of almost all cervical cancers [8,10]. Therefore, it is important to monitor abnormal results over time in order to detect early lesions, when possible.

In this study, we conducted a retrospective cohort review to compare the performance of HPV testing and cervical cytology in screening for high-grade squamous lesions, while also quantifying the frequency of high-risk HPV infection by genotype. The data were collected from all tested patients at our Oregon-based academic institution between October 2021 and June 2023. This study provides robust insights derived from the patients screened and followed up with in a routine clinical setting, offering a meaningful representation of real-world clinical practices.

## 2. Materials and Methods

### 2.1. Study Design and Data Analysis

This study was approved by the Institutional Review Board (IRB) at the Oregon Health & Science University (OHSU) under protocol #STUDY00025475. The study period spanned from October 2021 to June 2023. All patients between age 21 to 65 screened at OHSU for cervical cancer were eligible. The cases in which HPV testing was performed in other contexts, such as screening for anal squamous cell carcinoma, were excluded. Cytology, HPV testing, and the histological examination of cervical biopsies were performed and issued in final pathology reports. The tests were identified based on common procedure coding (CPT) systems related to cytology, HPV testing, and cervical biopsy.

Co-testing is defined as cases for which HPV PCR and cytology tests were ordered and conducted from the same collection. Otherwise, they were flagged as either HPV PCR only or cytology only. After the collections were identified, they were paired with biopsy results that occurred during the study period and a 6-month lookback window for cases with abnormal results before October 2021, when applicable. The HPV/cytology tests that occurred up to 6 months before the biopsy are included. If multiple collections or multiple biopsies occurred within the lookback period, each unique combination was represented in the data to comprehensively show how the testing matched with biopsy results. One patient can have multiple biopsy results in different categories.

### 2.2. Cytology Preparation and Interpretation

The cervical epithelium, collected using an endocervical brush/spatula, was placed in BD SurePath^TM^ (BD, Sparks, MD, USA) preservative fluid following the institutional standard operating procedure. The slides were processed with the Totalys^TM^ SlidePrep System (BD, Sparks, MD, USA) following the manufacturer’s instructions. The cytology results were reported using the 2014 Bethesda system for the classification of squamous lesions. The relevant categories and corresponding acronyms are as follows: negative for intraepithelial lesion (NIL), atypical squamous cells of uncertain significance (ASCUS), low-grade squamous intraepithelial lesion (LSIL), atypical squamous cells cannot exclude high-grade squamous intraepithelial lesion (ASC-H), and high-grade squamous intraepithelial lesion (HSIL) [11]. Glandular lesions including atypical glandular cells and adenocarcinoma were also included.

### 2.3. HPV Testing

Cobas^®^ HPV is a qualitative real-time PCR assay that detects fourteen high-risk HPV types: 16, 18, 31, 33, 35, 39, 45, 51, 52, 56, 58, 59, 66, and 68. The cervical epithelium, collected in BD SurePath^TM^ Preservative Fluid (BD, Sparks, MD, USA), was transported to the laboratory at room temperature for HPV testing on the Cobas^®^ 6800 and 8800 systems following the manufacturer’s instructions (Roche Diagnostics, Pleasanton, CA, USA). A minimum of 1.0 mL but no more than 4.0 mL of specimen in the preservation fluid was loaded into the machine for DNA extraction and PCR amplification. Human β-globin amplification and detection are included in Cobas^®^ HPV as an internal control to differentiate HPV-negative specimens from those that do not exhibit an HPV signal due to insufficient cell mass in the specimen. All HPV-negative specimens must have a valid β-globin signal within a pre-defined range to be identified as valid negatives. The results of positive or negative HPV and genotypes were reported to the laboratory informatics system (LIS).

### 2.4. Cervical Biopsy

Histological examinations of cervical biopsies, performed with or without colposcopy, including punch, cone, endocervical curettage, and loop electrosurgical excision procedure (LEEP) specimens were evaluated in our department by subspecialty trained gynecologic pathologists. The diagnosis categories include negative for intraepithelial lesion (NIL) and cervical intraepithelial neoplasia I (CIN 1), II (CIN 2) and III (CIN 3). Histologic CIN1 lesions correspond to LSIL cytology, while CIN 2 and CIN 3 lesions correspond to that of HSIL [12].

### 2.5. Statistical Analysis

All data analyses and visualization were performed in Tableau version 2023.3.1. (Salesforce, Inc., San Francisco, CA, USA) and SPSS version 29 (IBM, Armonk, NY, USA). Two proportion z tests were used and a *p* value < 0.05 is considered statistically significant.

## 3. Results

### 3.1. Characteristics of Patient Cohort

**Figure 1:**
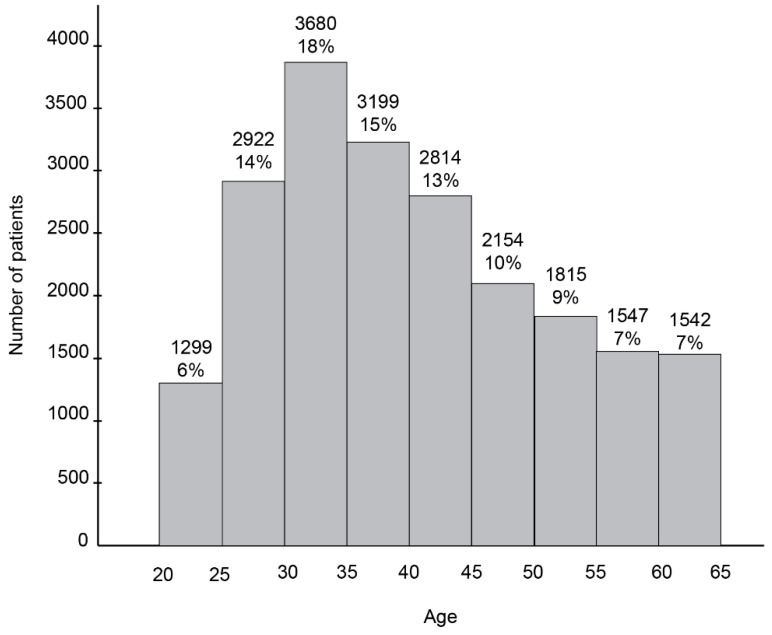
Age distribution of the study cohort.

A total of 20,972 individuals underwent testing during the 21-month study period, resulting in 22,488 total cases. Of these, 94.2% (19,761) completed one test, 4.8% (1012) completed two tests, and a small percentage (199, 0.95%) completed three or more tests. The age distribution of the study cohort is shown in Figure 1. The patients’ ages ranged from 21 to 65, with a median of 39 years. The largest age group included age 30 to 35 years (18%), followed by 35 to 39 years (15%), 25–29 years (14%) and 40–45 years (13%). Among the patients who completed only one test, 68% (13,450) underwent co-testing, while 21% (4122) and 11% (2189) completed cytology only or HPV testing only, respectively.

### 3.2. Overall Test Summary with Paired Biopsy Findings

Of all 22,488 cases, 15,424 (68.6%) tests involved HPV-cytology co-testing, 4353 (19.4%) were cytology-alone tests, and 2711 (12.0%) were HPV-alone tests (Table 1). Among these, 1162 (7.5%) tests showed abnormal cytology results, defined as any result on the squamous intraepithelial neoplasia spectrum, including ASCUS, LSIL, ASC-H and HSIL. A total of 1141 (7.4%) tests had abnormal HPV results, and 1065 (6.9%) tests produced abnormal results for both cytology and HPV. For tests with cytology alone, 178 out of 4353 tests (4.1%) yielded abnormal cytology results. When the HPV test was performed alone, 283 out of 2711 tests (10.4%) produced abnormal HPV results.

The data were further stratified to compare the results between co-testing, cytology, and HPV PCR testing with biopsy diagnoses. A total of 574 co-testing cases had paired biopsy results. Ten biopsies were performed with cytology tests alone and nine with HPV tests alone (Table 2). In the co-testing group for cytology results, 229 (39.9%) cases had normal cytology. The remaining 345 (60.1%) cases with abnormal cytology included 140 ASCUS, 113 LSIL, 40 ASC-H, 36 HSIL, and 16 atypical glandular cells cases. No adenocarcinoma was identified.

Among the 229 cases with normal cytology, approximately 65% (149 cases) had concordant benign biopsy results. The remaining 80 cases (34.9%) had various degrees of discordant results, with 44 cases (19.2%) showing normal cytology but CIN1/LSIL on biopsy, and 20 cases (8.7%) showing CIN2 on biopsy. Importantly, 16 cases (7.0%) were cytologically normal but diagnosed as CIN3/HSIL on biopsy. Among the cases diagnosed as CIN3 on biopsy, 82.9% had abnormal cytology (*N* = 94). The HSIL cytology diagnosis showed a good concordance rate with biopsy, with 31 (31/36, 86.1%) cases confirmed as CIN2 or CIN3 on biopsy. The diagnoses of ASCUS, ASC-H, and LSIL leaned toward a more varied biopsy diagnosis, ranging from benign to CIN3/HSIL. Two atypical glandular cell cases had HSIL at biopsy.

A chart review was performed on the 16 cytology-negative but biopsy-confirmed CIN3 cases, all of which were accompanied by high-risk HPV positivity. The cytology-negative results were accurately documented. Of the 16 patients, 7/16 (43.8%) had a history of persistent high-risk HPV or abnormal cytology (ASCUS), while 9/16 (56.2%) did not have prior documentation in our electronic health record system. However, based on the age range (35–57 years old), we suspected that these patients likely had prior abnormal screening results elsewhere, which may have prompted physicians to perform a biopsy.

In the co-testing group with HPV PCR results, 119 (20.7%) were HPV negative and 455 (79.3%) were HPV positive, with 266 having abnormal biopsy results. Within the 455 HPV-positive results, more than 60% of the cases were non-16/18 HPV types (283, 62.2%), followed by HPV 16 (104, 22.9%), and HPV 18 (33, 7.3%). The presence of co-infection with multiple HPV types is not rare. Twenty-six (5.7%) cases were positive for HPV 16 and one other high-risk strain, while nine (2.0%) were positive for HPV 18 and one other high-risk strain. The correlation of a HPV-negative status with benign biopsy diagnosis was good, with 92 cases (77.3%) being compatible among the 119 HPV-negative cases. However, seven HPV-negative cases showed CIN2 (5.9%), and nine tests (7.6%) indicated CIN3 on biopsy. Overall, the HPV-negative results in co-testing provided a 13.5% false reassurance. Approximately half of the cases with only HPV 16 or 18 positivity were negative for intraepithelial lesions on biopsy (52/104 cases, 50% vs. 18/33cases, 54.5%, respectively), while 27.9% (29/104 cases) and 15.2% (5/33 cases) had CIN3/HSIL on biopsy in each corresponding group. Importantly, of the cases infected by non-HPV16/18 high-risk HPV, 48 (17.0%) had CIN3/HSIL in biopsy specimens. Among the cases diagnosed as CIN3 on biopsy, 90.6% had a positive HPV testing (*N* = 96). The combination infection of HPV 16 or 18 along with other high-risk types did not put patients at a higher risk of CIN3/HSIL, and the diagnosis results of the biopsies varied among the categories from benign to CIN3/HSIL.

A subset of 133 cases had a positive diagnosis in both HPV and cytology combined. In total, 99 cases were HPV positive with a cytology LSIL diagnosis, while 34 cases were HPV positive with an HSIL diagnosis in cytology. The HPV+/LSIL+ cytology group had 30.3% (*n* = 30) of cases of benign biopsy, 33.3% (*n* = 33) of CIN1/LSIL, 19.2% (*n* = 19) of CIN2, and 17.2% of CIN3/HSIL. In contrast, only 5.9% (*n* = 2) of cases with HPV+/HSIL+ cytology had negative biopsies. Two-thirds (*n* = 23, 67.6%) of the cases had concordant CIN3/HSIL biopsies.

In addition to the cases performed with co-testing, a small number of cases had a cytology alone (*n* = 10) or HPV testing alone (*n* = 9) before proceeding to biopsy. Of the cases with only cytology prior to biopsy, three had cytologic findings that were negative for intraepithelial lesion (NIL); all three were also NIL on biopsy. Another three had cytologic findings of ASCUS, with more varied biopsy results ranging from normal to CIN3. The last four cases had cytologic findings of LSIL; three were NIL on biopsy, and the final case was found to have CIN1. No diagnosis of ASC-H, HSIL atypical glandular cells, or adenocarcinoma were reported. Among the nine cases with HPV testing only with a subsequent biopsy, four cases were HPV negative, one case was positive for HPV 18, and the remaining four were positive for non-16/18 high-risk HPV. All five positive cases had negative biopsies (NIL). Of the four negative cases, two had negative biopsies (NIL) and two were found to have HSIL.

The overall observation from our dataset suggests that in co-testing, both the HPV and cytology methods missed about 7% of patients with high-grade CIN3/HSIL lesions on biopsy (9/119 vs. 16/229). Nearly half of the cases with a HPV16- or HPV18-positive PCR had benign biopsy results. High-risk HPV strains other than HPV16/18 comprised half of the positive HPV cases, and 17% had CIN3/HSIL on biopsy.

### 3.3. Comparison of Cytology and HPV Genotyping in Detecting CIN2 and CIN3 Lesion

The detection of high-grade dysplasia early is of critical importance for cervical cancer screening. To compare the sensitivity and specificity of cytology and HPV genotyping in detecting CIN2 and CIN3 lesions, we grouped the biopsy results into three categories: CIN2/HSIL, CIN3/HSIL, and negative for CIN3/HSIL, which included CIN1 and NIL. A total of 584 cases including co-testing and cytology alone from Table 2 were included (Table 3). For the detection of CIN2 and CIN3 combined, cytology had a sensitivity of 78.3% and a specificity of 48.9%. The PPV was 39.6%, and the negative predictive value (NPV) was 84.0%.

Similarly, HPV status was categorized into three groups to evaluate CIN2 and CIN3 lesions. A total of 591 cases from co-testing and PCR alone, with paired biopsy tests, were included (Table 4). The number is slightly higher than that from cytology, as individuals may have undergone multiple biopsies and are counted separately when CIN2 or CIN3 was reported. For the detection of CIN2+, HPV testing had a sensitivity of 88.8% and a specificity of 27.1%. The PPV was 34.4%, and the NPV was 84.8%.

Overall, HPV testing showed a slightly higher sensitivity than cytology (88.8% vs. 78.3%, *p* = 0.128), although the difference is not statistically significant. The specificity for detecting CIN2/3 lesions is significantly higher in cytology than HPV (48.9% vs. 27.1%, *p* < 0.01).

## 4. Discussion

While both the ACS and ASCCP guidelines recommend HPV-based testing as the primary screening method for cervical cancer, concerns remain about its use as the only screening modality. This is particularly true in countries with a low HPV vaccine rate and without socialized healthcare systems, like the United States, where the data on HPV primary screening remains limited. The American Society of Cytopathology still recommends HPV and cytology co-testing over HPV testing alone as the optimal method for primary screening [2,13]. Our paper adds to the depth and breadth of knowledge on HPV testing in cervical cancer screening within real-world scenarios at a large academic institution.

In this retrospective study conducted at an academic hospital in Oregon over a 21-month period, we analyzed a total of 22,488 laboratory tests from 20,972 individuals aged 21–65 undergoing cervical cancer screening. Most cases (~70%) performed co-testing in our hospital. Within this group, abnormal cytology, abnormal HPV testing, or abnormalities in both were detected in 7.5%, 7.4%, and 6.9% of the cases, respectively. In contrast, among the cases where only cytology was performed, 4.1% showed abnormalities. These findings align closely with those from the ATHENA study, which included 47,208 women aged 21 years and older, estimating similar rates of cytologic abnormalities and hrHPV positivity [14]. Our study also found that hrHPV positivity rates ranged from 7.4% to 10.4%, depending on whether co-testing or HPV testing alone was conducted. This is consistent with findings from the ATHENA study and an HPV prevalence study conducted in the National Health and Nutrition Examination Survey (NHANES) [15]. The overall hrHPV prevalence in the ATHENA and NHANES studies was 12.6% and 15.2%, respectively. Importantly, in the co-testing group, there were cases that were negative in both testing modalities yet still missed diagnoses of CIN2 and CIN3 at biopsy, with cytology overlooking 20 cases of CIN2 and 16 cases of CIN3, while HPV testing missed 7 cases of CIN2 and 9 cases of CIN3. HPV testing alone also missed two cases of CIN3, although the sample size for this group is small. An additional chart review of the 16 cytology-negative but biopsy-confirmed CIN3 cases confirmed that all patients had concurrent HPV positivity. In total, 7 of the 16 (43.8%) had a history of persistent high-risk HPV or abnormal cytology (ASCUS) in the past, while 9 out of 16 (56.2%) did not have prior documentation in our EHR. The age range of the group spans from 35 to 57 years, suggesting that these patients are less likely to undergo initial screening. Therefore, the percentage (7.0%) of cytology-normal and biopsy-confirmed CIN3 in our study is higher than the reported 4.5% immediate CIN3+ risk in HPV-positive ASCUS in the initial screening group, as stated in the 2019 ASCCP Guidelines [3].

Despite the cases where CIN2 and CIN3 were missed, our analysis of the sub-cohort with biopsy results and HPV/cytology findings showed the higher sensitivity of HPV testing compared to cytology in detecting high-grade lesions (88.8% vs. 78.3%). These overall observations highlight the inherent limitations of screening methods and underscore the importance of regular screening. Studies from China and Japan also emphasize the varying effectiveness and implications of screening methods in different populations, reinforcing the argument for the wider adoption of HPV primary screening based on its demonstrated efficacy and lower referral rates for unnecessary procedures compared to cytology alone [16,17].

Regarding screening sensitivity and specificity, a large cohort study performed at Kaiser Permanente Northern California (KPNC) highlighted the higher specificity of cytology in immediate cancer risk assessment. However, its lower sensitivity and negative predictive value compared to HPV testing decreases its applicability in long-term risk prediction [18]. The data from four large-scale European randomized control trials have demonstrated that HPV-based screening provides 60–70% greater protection against invasive cervical carcinomas compared with cytology [19]. While our findings do not conclusively demonstrate HPV testing’s significantly higher sensitivity due to our smaller cohort size, the study does reveal cytology’s superior specificity for detecting high-grade lesions compared to HPV testing. Concerns about negative HPV testing in CIN3/HSIL lesions persist, although our observed rate of CIN3+/HPV- cases (6.99%) was lower than that reported in the literature (8.3–14%) [20,21].

HPV testing’s high sensitivity and the U.S. Food and Drug Administration (FDA)-approved high-throughput HPV testing systems make it an attractive option for cervical cancer screening. Automated molecular testing offers operational advantages over labor-intensive cytologic screening, particularly in managing the high volume of routine pap smears, many of which yield negative results. The recent FDA approval of primary HPV self-collection for screening is a significant step toward reducing barriers to sample collection and increasing screening accessibility [22]. This advancement is particularly crucial as persistent disparities in cervical cancer incidence exist across race/ethnicity and socioeconomic status. While the overall squamous cell carcinoma incidence has declined in most racial/ethnic groups, the rates among non-Hispanic whites have remained stable [23]. The incidence rate of adenocarcinoma is notably elevated among non-Hispanic whites, particularly in the 40–59 years age group. This disparity underscores the need to enhance access and adherence to recommended screening practices for primary and secondary prevention [24]. By improving access to innovative screening methods as well as high-throughput molecular testing, we can strive towards a more equitable healthcare system and reduce the burden of cervical cancer for all populations.

HPV has been implicated in nearly all cervical squamous cell carcinoma cases worldwide [25]. Notably, HPV 16 and 18 are responsible for over 70% of cervical cancers, with several other high-risk genotypes also contributing significantly [26,27,28]. Our study demonstrated that HPV 16 was identified in 12.5% of CIN1 cases, 9.6% of CIN2 cases, and 27.9% of CIN3 cases. The HPV 16-positive rate in CIN1 is very similar to the published data from the ATHENA study and other meta-analysis data, which were 12.8% and 18.7%, respectively [14,29]. However, the positive rate in higher CINs was much smaller than the previous reported data, at 29.7% of CIN2 and 51.2% of CIN3 in the ATHENA study and 45.3% of CIN2/3 combined cases in a pooled study [14,30]. This lower percentage could be attributed to the relatively smaller sample size from our study (574 vs. 1178 in the ATHENA study).

Regarding HPV genotypes, the high prevalence of non-HPV16/18 high-risk genotypes is also significant. In our cohort, nearly half of the HPV-positive tests (49.3%) of the co-testing with biopsy group were non-HPV16/18 types, 17% of which had a paired CIN3/HSIL biopsy. Of all the cases with CIN3/HSIL, 50% had a high-risk strain other than HPV 16 or 18. While we are still evaluating the vaccination status of our cohort, it is important to keep in mind that HPV vaccination might shift the prevalence of hrHPV among the population. A population study conducted in Denmark has clearly shown that among the 6233 screening samples from women aged 23, the vaccine HPV 16/18 strains were almost eliminated, with only a 0.9% prevalence, while the prevalence of non-vaccine hrHPV types remained constant (about 34%) since the initiation of the HPV vaccination initiative in October 2008 [31]. Our study underscores the importance of broad HPV testing matrices, beyond HPV16/18 alone, despite the FDA-approved options focusing primarily on these genotypes. Multiple HPV genotype co-positivity was observed in 3.2% of cases. Previous studies have shown that 20–58% of HPV-positive women were infected with multiple HPV genotypes [30,32]. Multiple infections tend to be found more frequently in young women or women with an impaired immune response [33,34].

There are several limitations to this study. The relatively small cohort size, compared to larger clinical trial cohorts, is one such limitation. Additionally, a major challenge with real-world data is that patients may only stay within the healthcare system for a short period of time, making it difficult to obtain a complete medical history. This complicates the ability to appropriately assess the risk for CIN3+. Despite these limitations, the data presented here offer a valuable reference for understanding the real-world applicability and limitations of HPV and cytology testing for CIN3+ detection. In summary, this study retrospectively evaluated different test modalities for cervical cancer screening in Oregon. Our data confirmed that HPV testing has slightly higher sensitivity but is less specific than cytology in detecting CIN2 and CIN3 high-grade lesions. Additionally, the automation of HPV genotyping offers operational advantages in the laboratory. In line with recent screening guideline updates, HPV testing has demonstrated its efficacy in screening.

## Figures and Tables

**Table 1:** Overall testing results.

Test Types	Test Results		Number of Tests	Percentages of Each Test Type
Co-testing	Normal		12,056	78.2%
	Abnormal	Cytology only	1162	7.5%
		HPV only	1141	7.4%
		Cytology and HPV	1065	6.9%
**Subtotal**			15,424	
Cytology alone	Normal		4175	95.9%
	Abnormal		178	4.1%
**Subtotal**			4353	
HPV alone	Normal		2428	89.6%
	Abnormal		283	10.4%
**Subtotal**			2711	
**Grand total**			22,488	

**Table 2:** Co-testing, cytology-alone or PCR-alone tests with paired biopsy diagnosis.

Result Category	Diagnosis	Number of Tests	Biopsy Diagnosis				
			NILM	CIN1/LSIL	CIN2/HSIL	CIN3/HSIL	Subtotal per Row *
**Co-Testing**							
Cytology	NILM	*N* = 574	149 (65.1%)	44 (19.2%)	20 (8.7%)	16 (7.0%)	*N* = 229
	ASCUS		67 (47.8%)	32 (22.9%)	22 (15.7%)	19 (13.6%)	*N* = 140
	LSIL		41 (36.3%)	36 (31.9%)	19 (16.8%)	17 (15.0%)	*N* = 113
	ASC-H		12 (30.0%)	3 (7.5%)	10 (25.0%)	15 (37.5)	*N* = 40
	HSIL		2 (5.6%)	3 (8.3%)	6 (16.7%)	25 (69.4%)	*N* = 36
	Adenocarcinoma		0	0	0	0	*N* = 0
	Atypical glandular cells		9 (69.2%)	1 (7.7%)	1 (7.7%)	2 (15.4%)	*N* = 13
	Atypical glandular cells, favor neoplastic		2 (66.7%)	1 (33.3%)	0	0	*N* = 3
			Subtotal per column				
			*N* = 282	*N* = 120	*N* = 78	*N* = 94	
HPV status	HPV -	*N* = 574	92 (77.3%)	11 (9.2%)	7 (5.9%)	9 (7.6%)	*N* = 119
	HPV 16+		52 (50.0%)	13 (12.5%)	10 (9.6%)	29 (27.9%)	*N* = 104
	HPV 18+		18 (54.5%)	7 (21.2%)	3 (9.1%)	5 (15.2%)	*N* = 33
	HPV other +		107 (37.8%)	77 (27.2%)	51 (18.0%)	48 (17.0%)	*N* = 283
	HPV 16 and other +		9 (34.6%)	6 (23.1%)	6 (23.1%)	5 (19.2%)	*N* = 26
	HPV 18 and other +		3 (33.3%)	5 (55.6%)	1 (11.1%)	0	*N* = 9
			Subtotal per column				
			*N* = 281	*N* = 119	*N* = 78	*N* = 96	
Combined	HPV+ and LSIL+	*N* = 133	30 (30.3%)	33 (33.3%)	19 (19.2%)	17 (17.2%)	99
	HPV+ and HSIL+		2 (5.9%)	3 (8.8%)	6 (17.6%)	23 (67.6%)	34
**Cytology alone**							
Cytology	NILM	*N* = 10	3 (100%)	0	0	0	3
	ASCUS		1 (33.3%)	0	1 (33.3%)	1 (33.3%)	3
	LSIL		3 (75.0%)	1 (25.0%)	0	0	4
	ASC-H		0	0	0	0	0
	HSIL		0	0	0	0	0
	Adenocarcinoma or atypical glandular cells		0	0	0	0	0
**HPV alone**							
HPV status	HPV -	*N* = 9	2 (50%)	0	0	2 (50%)	4
	HPV 18+		1 (100%)	0	0	0	1
	HPV other +		4 (100%)	0	0	0	4

* The subtotal is the sum of cases per row. The percentage is calculated by the number of cases divided by the subtotal. NILM = negative for intraepithelial malignancy; ASCUS = atypical squamous cells of undetermined significance; ASC-H = atypical squamous cells, cannot exclude high-grade squamous intraepithelial lesion; CIN = cervical intraepithelial neoplasia; LSIL = low-grade squamous intraepithelial lesion; and HSIL = high-grade squamous intraepithelial lesion.

**Table 3:** Sensitivity and specificity of cytology diagnosis with paired CIN2/HSIL or CIN3/HSIL biopsy (*n* = 584).

		Biopsy		
		Negative for CIN2/HSIL or CIN3/HSIL	CIN2/HSIL	CIN3/HSIL
Cytology	Normal	200 (34.2%)	20 (3.4%)	18 (3.1%)
	Abnormal *	209 (35.8%)	58 (9.9%)	79 (13.5%)
Sensitivity	78.3%			
Specificity	48.9%			
PPV	39.6%			
NPV	84.0%			

* An abnormal cytology is defined as any result other than negative for intraepithelial lesions.

**Table 4:** Sensitivity and specificity of HPV status with paired CIN2/HSIL or CIN3/HSIL biopsy (*n* = 591).

		Biopsy		
		Negative for CIN2/HSIL or CIN3/HSIL	CIN2/HSIL	CIN3/HSIL
HPV status	Negative	112 (19.0%)	8 (1.4%)	12 (2.0%)
	Positive	301 (50.9%)	71 (12.0%)	87 (14.7%)
Sensitivity	88.8%			
Specificity	27.1%			
PPV	34.4%			
NPV	84.8%			

## Data Availability

The data are not publicly available due to privacy, and the data presented in this study are available upon request from the corresponding author.
